# Engineered tunneling layer with enhanced impact ionization for detection improvement in graphene/silicon heterojunction photodetectors

**DOI:** 10.1038/s41377-021-00553-2

**Published:** 2021-05-31

**Authors:** Jun Yin, Lian Liu, Yashu Zang, Anni Ying, Wenjie Hui, Shusen Jiang, Chunquan Zhang, Tzuyi Yang, Yu-Lun Chueh, Jing Li, Junyong Kang

**Affiliations:** 1 Collaborative Innovation Center for Optoelectronic Semiconductors and Efficient Devices, Pen-Tung Sah Institute of Micro-Nano Science and Technology/ Department of Physics, Xiamen University, Xiamen, 361005 China; 2 San’an Optoelectronics Co., Ltd., Xiamen, 361005 China; 3 Department of Materials Science and Engineering, Tsing Hua University, Hsinchu, 30013 China

**Keywords:** Electronic properties and devices, Optoelectronic devices and components

## Abstract

Here, an engineered tunneling layer enhanced photocurrent multiplication through the impact ionization effect was proposed and experimentally demonstrated on the graphene/silicon heterojunction photodetectors. With considering the suitable band structure of the insulation material and their special defect states, an atomic layer deposition (ALD) prepared wide-bandgap insulating (WBI) layer of AlN was introduced into the interface of graphene/silicon heterojunction. The promoted tunneling process from this designed structure demonstrated that can effectively help the impact ionization with photogain not only for the regular minority carriers from silicon, but also for the novel hot carries from graphene. As a result, significantly enhanced photocurrent as well as simultaneously decreased dark current about one order were accomplished in this graphene/insulation/silicon (GIS) heterojunction devices with the optimized AlN thickness of ~15 nm compared to the conventional graphene/silicon (GS) devices. Specifically, at the reverse bias of −10 V, a 3.96-A W^−1^ responsivity with the photogain of ~5.8 for the peak response under 850-nm light illumination, and a 1.03-A W^−1^ responsivity with ∼3.5 photogain under the 365 nm ultraviolet (UV) illumination were realized, which are even remarkably higher than those in GIS devices with either Al_2_O_3_ or the commonly employed SiO_2_ insulation layers. This work demonstrates a universal strategy to fabricate broadband, low-cost and high-performance photo-detecting devices towards the graphene-silicon optoelectronic integration.

## Introduction

Benefited from the series of excellent electrical and optical properties, such as broadband absorption, high carrier mobility, high carrier concentration, and good transparency^1^, graphene demonstrates attractive applications in high-performance photodetectors with excellent broadband operation and ultra-fast response^1–3^. Currently, the metal-graphene-metal (MGM) structure^2^, graphene double-layer heterostructure^4^, and graphene/silicon (GS) heterostructure^5,6^ are the most adopted device models presenting their individual advantages. Among these device types, the GS heterojunction Schottky photodiode exhibits the most promising applications to the graphene integrated silicon photonics^7–10^, due to its prominent rectification behavior, low dark current, good stability, and high photo-responsivity^7,11–14^. The up-to-date report has demonstrated the perfect photo-responsivity of as high as 0.73 A W^−1^ in the GS heterojunction photodetectors exceeding the conventional silicon-based PIN devices^6^. By further optimizing the band structure using the ultra-shallow junction, ultraviolet (UV) enhanced photodetection was also realized in this kind of Schottky type devices^15^. Therefore, it can be claimed that the GS heterojunction photodetectors are presenting the comparable detecting ability to that of state-of-the-art silicon-based devices, besides their superior advantages of low-cost and easy integration with silicon technologies.

However, in spite of the high infrared light response originated from the narrow band gap of the silicon substrate (~1.1 eV), which acts as the active layer of the photodetectors, the UV photoresponsivity of this type of devices is still weak due to the high reflection and limited penetration depth of UV light (*λ* < 400 nm) in silicon^16^. What’s more, the leakage current induced by the surface states within the GS interface generally results in a low specific detectivity^6^. With the aim to address these issues, interface engineering, such as using the tunneling heterostructures^17–19^, introducing the passivation layer^20–22^, or modification with quantum dots/nanoparticles^23,24^, has been adopted to improve the photo-detection in the GS heterojunction photodetectors. In addition, the integrating of plasmonic nanostructures also has been demonstrated to be an effective strategy to improve the detecting performance via the modified absorption and internal photoemission process^25,26^. While for the tunneling structure, which is usually constructed by inserting an insulating layer into metal-semiconductor (MS) interface, it has been considered to be the most attractive method to obviously increase the detectivity with the much easily constructed device structures^27,28^. This functionalized layer not only can serve as an interface passivation material to inhibit the static charges’ transfer^29,30^, which is similar to that used in other strategies including the integration of plasmonics and quantum dots, but also can potentially enable the impact ionization resulting in a significant increase in the photocurrent multiplication^29,31^. In addition, an enhanced response speed also can be expected in the tunneling heterostructure because of the dominated ultra-fast quantum tunneling process rather than by the drift in the depletion region for the carriers’ transfering^18^. However, since the photocurrent in tunneling structures is mainly determined by the applied electric field on the insulator layer and the barriers’ hight^31^, the band alignment of the tunneling junction and the thickness control of the tunneling layer are very critical. On the other hand, the novel photogain *via* impact ionization during the tunneling process also shows a strong dependence on the specific insulating material^32,33^. Therefore, the effective design and fabrication of the tunneling structure are still challenging in GS heterojunction photodetectors in order to achieve high responsivity and detectivity beyond the traditional silicon-based detectors.

In this work, the tunneling process engineering was proposed by introducing the atomic layer deposition (ALD) deposited wide-bandgap insulating (WBI) layer between the graphene/semiconductor interface to manipulate the photo-induced carriers’ transportation with the aim to achieve an enhanced photocurrent multiplication via the impact ionization. According to the results, the introduced insulating layer has effectively suppressed the dark current of the device while the photocurrent was greatly improved under a reverse bias at the same time. The impact ionization both for the hot carriers from graphene and minority carriers from silicon within the tunneling layer was considered to contribute the obvious photocurrent multiplication, yielding the photo-gain up to 3.5 under the 365-nm UV illumination at a bias of −10 V in this device structure. A broad spectral response enhancement in this tunneling photodetector also was demonstrated to be especially significant between 760 and 900 nm, and the peak responsivity achieved 5.8-times photogain under the 850 nm illumination. The work experimentally demonstrates an effective strategy for improving detectivity in the GS heterostructure devices, paving the way towards the next-generation high performance, low-cost and integratable photodetectors targeting at a broad spectrum or specific wavelength applications.

## Results

**Fig. 1:**
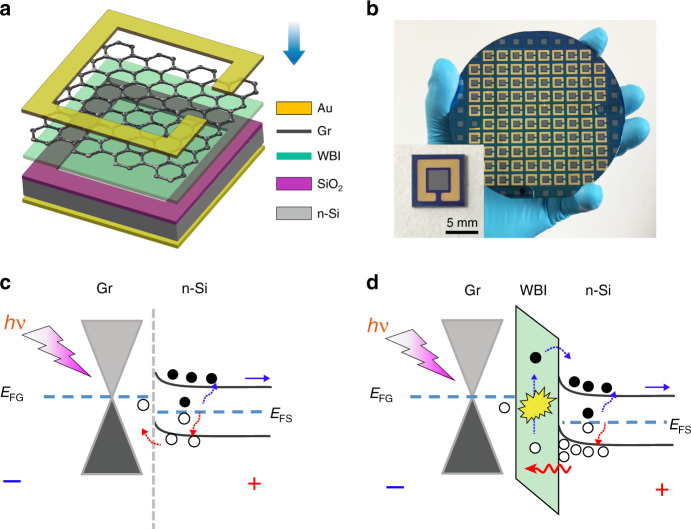
Device structure and proposed working mechanism for the devices. **a** Schematic and **b** photo image of the fabricated wafer-scale graphene/insulator/Si (GIS) heterojunction photodetectors. The device size was 8 × 8 mm^2^ with a center active region of 3 × 3 mm^2^. **c**, **d** Shows the corresponding energy band diagram of the conventional GS heterojunction photodetector and the GIS heterojunction photodetector under reverse bias and illumination, respectively.

The device structure and energy-band diagram of the graphene/insulator/silicon (GIS) heterojunction photodetectors were schematically shown in Fig. 1. In this device (Fig. 1a and b), a 3 mm × 3 mm silicon window on the SiO_2_/n-Si substrate was fabricated through the lithography, followed by the insulation layer deposition, 3–5 layers’ graphene transferring, and contact metal’s deposition (Fig. S1, Supporting Information). The Raman spectra indicate the well-maintained quality for the transferred graphene on silicon with the typical characteristics of few layers (Fig. S2). For the conventional GS heterostructure photodetector (Fig. 1c), due to the different work functions between graphene and n-type silicon, Schottky junction is formed in the interface of graphene/n-Si^7,34^. Under light illumination, the optical absorption mainly takes place in silicon substrate, while graphene mainly acts as a transparent electrode due to its high carrier mobility and high transparency. And the photo-generated carriers can be separated by the built-in electric field: the holes move to top electrode through graphene while the electrons move to the bottom electrode through silicon. After introducing a thin WBI layer between the graphene and silicon (Fig. 1d), the tunneling structure would be formed and the energy-band diagram of the photodetector under reverse bias would be aligned as shown in Fig. 1d. In this case, the dark current is expected to be suppressed due to the increased Schottky barrier height (SBH), and the photo-generated excess holes would accumulate at the insulation-semiconductor interface. Further, if the insulating layer is thin enough for carriers’ quantum tunneling, the impact ionization driven by high electric felid would subsequently occur to realize the photocurrent multiplication^28^.

**Fig. 2:**
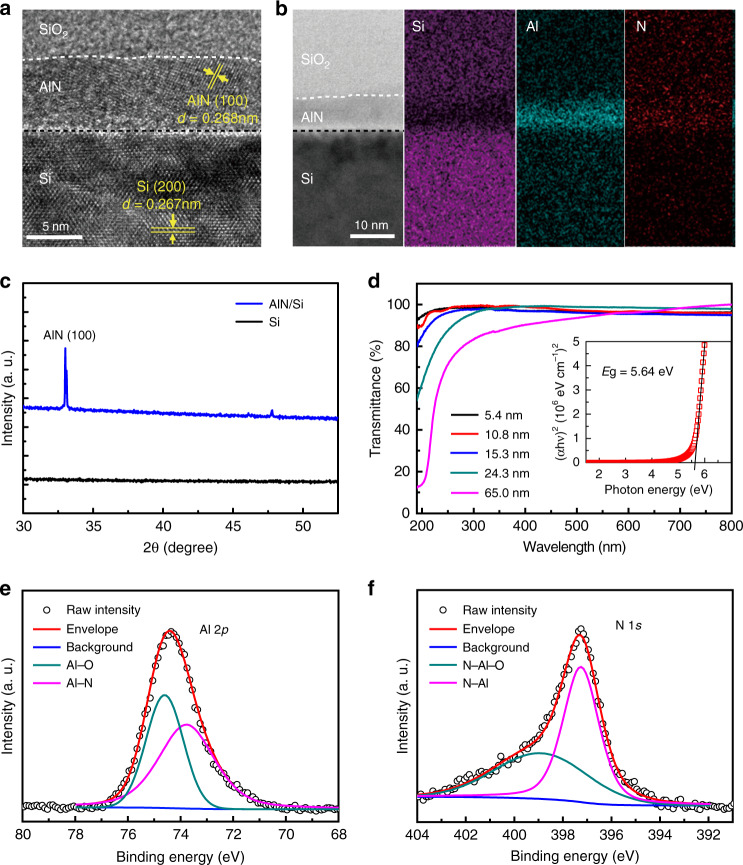
Morphology, crystallinity, and optical characterization of the AlN film. **a** Cross-sectional HR-TEM image of the ALD deposited AlN films on Si substrate and **b** the corresponding EDX elemental mappings. **c** XRD patterns of the bare Si substrate and 65 nm AlN film grown on Si. **d** Optical transmission spectra of AlN films grown on sapphire substrates with different thicknesses. The Tauc plot for the estimation of the bandgap of AlN was shown in inset. **e**, **f** Shows the XPS spectra for Al 2*p* and N 1*s* of the AlN film, respectively.

In order to demonstrate the proposed tunneling effect enhanced photo-responsivity by introducing the wide-bandgap tunneling layer, aluminum nitride (AlN) was selectively used as the interlayer material firstly. Here, the AlN film used in this work was grown by ALD at a temperature of 380 °C and the detailed deposition parameters are shown in Fig. S3. The thickness of the AlN films was determined by the growth parameter of about 0.09-nm-thick per cycle (Table S1, Supporting Information). The scanning electron microscope (SEM) images of the AlN films before and after deposition on Si substrate can be seen in Fig. S4. Figure 2a shows the cross-section image of the SiO_2_ protected AlN/Si structure by the high-resolution transmission electron microscopy (HR-TEM), in which polycrystalline characteristics can be well resolved for the AlN layer. The interplanar spacing in AlN and Si grains were measured to be 0.268 nm and 0.267 nm, respectively, which correspond to the AlN (100) and Si (200) lattice structures. Further X-ray diffraction (XRD) analysis on the 65-nm AlN film grown on Si substrate also inferred the existence of AlN (100) plane with the main diffraction peak resolved at 33.1°. The transmission spectra shown in Fig. 2d indicated that the deposited AlN films have a satisfied crystal quality with a good optical transmittance. Here, the estimated band-edge (*E*_g_) by using the Tauc’s method^35^ from the absorption spectra was about 5.64 eV, showing a less value than the bulk AlN^36^. Understandably, kinds of crystal defects in the ALD deposited AlN film were the main reasons. Further XPS measurement well indicated the typical O related defects (Al–O bond at binding energy of 74.6 eV and N-Al-O bond at a binding energy of 398.8 eV) for the ALD deposited AlN film, which shows similar results as that reported in the previous work^37,38^, as shown in Fig. 2e and f for the Al 2*p* and N 1*s* spectra. It is believed that these defects would contribute to the carriers’ tunneling as well as the impact ionization, and this will be discussed later.

**Fig. 3:**
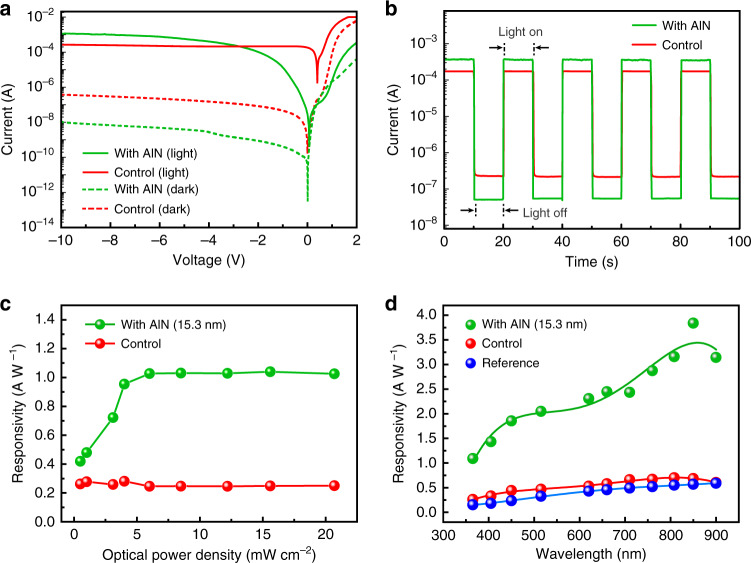
Photo-detection performance of the GIS devices. **a** The *I-V* characterizations of GIS tunneling photodetector with 15.3-nm AlN comparing with the conventional graphene/n-Si heterojunction photodetector under dark and light illumination. **b** Time-dependent photo-response for the GIS device under −10 V and 365-nm light illumination (12.2 mW cm^−2^). **c** Optical power-dependent responsivity in the GIS tunneling photodetector with the comparison to the control device without tunneling layer under 365-nm light illumination at a bias of −10 V. **d** Spectra-dependent photocurrent responsivity of the GIS tunneling photodetector with 15.3-nm AlN tunneling layer at a bias of −10 V, comparing with the control and reference devices.

The photodetection performances in the proposed tunneling devices were firstly evaluated by the current-voltage (*I-V*) characterization under the dark and light illumination (365 nm) conditions, as shown in Fig. 3a, with the comparison to the conventional Schottky device. It can be seen that due to the Schottky barrier, both the GIS devices with AlN and GS control photodetectors work as a usual rectifier diode under dark condition. However, as proposed above, the inserted AlN film served as a perfect barrier layer to significantly suppress the dark current of the device, showing at least one order of magnitude decrement at the bias of −10 V.

While under the light illumination, for the conventional GS photodetector, a light-induced photocurrent with a current saturation region can be observed in the reverse bias. In this conventional case, the photocurrent mainly depends on the incident light just as that in a typical Schottky photodiode, during which the photo-generated minority holes move to graphene induced by the built-in electrical field or the applied reverse bias while the majority electrons pass through the depletion region to generate the photocurrent. However, when a thin AlN film was inserted, carriers tunneling occurs in GIS photodetector by the applied reverse voltage providing the driving force for the carriers’ multiplication. Thus, significantly enhanced photocurrent was observed under the reverse bias over −2.8 V. Understandably, the formed barriers would suppress the carriers’ transport when the bias is in the forward bias case, resulting the lowered photocurrent comparing with the control GS device. The time-related photo-response shown in Fig. 3b clearly demonstrated the tunneling-effect induced prominent characteristics in the GIS device: much stronger photocurrent and lower dark current were obtained showing a significantly increased detectivity.

The photo responsivity under specific incident optical power was calculated using the equation *R* = (*I*_p_-*I*_d_)/*P*, where *I*_p_ is the photocurrent, *I*_d_ is the dark current and *P* is the applied optical power. The calculated specific responsivity and detectivity for the typical device under the UV light (365 nm) was 1.03 A W^−1^ and 2.94 × 10^7^ Jones with relative low NEP value of ~1.02 × 10^−8^ W Hz^−1/2^. Additional responsivity metrics for a batch of devices shown that average values of 1.02 ± 0.08 A W^−1^ and 0.22 ± 0.10 A W^−1^ were obtained for the GIS and control GS devices, respectively, indicating the satisfied uniformity in device performance (Fig. S5). The reverse bias dependent responsivity (Fig. S6) well reveals the avalanche multiplication characteristics where the applied electric-field (bias) across the AlN layer dominated impact ionization^39^. Here, the photocurrent multiplication factor (*M*_ph_) as a function of reverse bias (*V*) has been estimated by *M*_ph_ = [*I*_ph_ (V)− *I*_dark_ (V)]/[*I*_ph(unity)_−*I*_dark(unity)_] (Fig. S7), where *I*_ph_ (*V*) and *I*_dark_ (*V*) are multiplied photo- and dark currents, respectively; *I*_ph(unity)_ and *I*_dark(unity)_ are primary (unmultiplied) photo- and dark currents at the unity gain region^39,40^. And the unity photocurrent and dark currents were evaluated using the photocurrent at bias of −2.8 V of which the photocurrent for GIS device surpass the conventional GS Schottky photodetector. At this bias (−2.8 V), the calculated corresponding critical electric field for AlN multiplication layer is about 1.8 MV cm^−1^, which is consistent with the reported operation electrical field of 1.9 MV cm^−1^ for AlN avalanche multiplication^39^. With a trade of the responsivity, detectivity and ON/OFF ratio along with the reverse voltage (Figs. S6 and S7), the bias of −10 V has been used for the investigation.

In order to further understand the thickness-dependent tunneling effect of the inserting layer and achieve the maximum improvement, the photo-responses in the tunneling structures with different thicknesses of the AlN films were characterized with comparing to the control one, as shown in Fig. S8. It can be seen that with increasing the thickness of AlN, the *I-V* characteristics of the devices gradually changed from typical Schottky photodiode to the Schottky tunneling photodiode (Fig. S8a)^33,41^. The enlarged view of the photocurrent under reverse bias was shown in Fig. S8b, and the photocurrent value of the devices under the -10 V bias with the AlN-thickness increased was statistically plotted in inset. It can be inferred that the thickness of about 15 nm was the optimized one to achieve the best performance in the corresponding devices, while the too thin AlN (5.4 nm) could not form the effective tunneling distance to realize the multiplication obviously and the thicker AlN (24.3 nm) would further suppress the tunneling current.

The photo-response characteristics for the optimized GIS device with 15.3-nm AlN under the bias of −10 V and 365-nm light illumination with different light powers was shown in Fig. S9, comparing to the control device without AlN inserting layer. The obtained optical power-dependent responsivity was plotted in Fig. 3c. It can be seen that the photocurrent for the conventional GS photodetector is approximately linear with the incident light power, similar as a typical Schottky type photodetector^34,42^. The calculated responsivity shows a higher value in the weak incident light region of μW level and a relatively stable value about 0.25 A W^−1^ as the incident light power increased from μW to mW. However, in the GIS structure photodetector the responsivity initially increased as the light intensity rising and then reached a relatively stable value of ~1.03 A W^−1^ at the illumination power larger than ~5 mW cm^−2^. Comparing with the as-fabricated conventional GS heterojunction photodetectors and typical commercial silicon PIN photodetectors (Hamamatsu S2836-44K), respective ~4.2 and ~7.2 times enhancements in photo-responsivity were facilely achieved in the GIS devices. It should be noted that due to incident light power-dependent response feature, the linear dynamic range (LDR) performed on this GIS device has not been increased obviously compared to the control GS device (72.5 db vs 36.1 db), where LDR is defined by equation LDR = 20 log (I^*^_ph_/*I*_dark_)^43^, and I^*^_ph_ is the photocurrent measured at an incident optical power of 1 mW cm^−2^.

As shown in Fig. 3d, a broad spectral response enhancement also can be well recognized due to the tunneling effect induced optical gain in the optimized GIS device with 15.3-nm AlN at a reverse bias of −10 V. The peak optical responsivity was realized at the wavelength around 850 nm as well acknowledged in Si structure. Further optical power-dependent photo-response at 850 nm as shown in Fig. S10 demonstrates the similar feature that the responsivity achieves a higher and stable value when the incident optical power is increased to mW level. A peak optical responsivity of 3.96 A W^−1^ and detectivity of 1.13 × 10^8^ Jones was facilely obtained on the GIS device, presenting a 5.8-times photogain comparing with the control device in the conventional structure. Comparing with the recent reported GS herterojuntion photodetectors with an interface insulating layer (Table S2), the proposed tunneling structure in this work enables the devices holding a competitive detection performance.

**Fig. 4:**
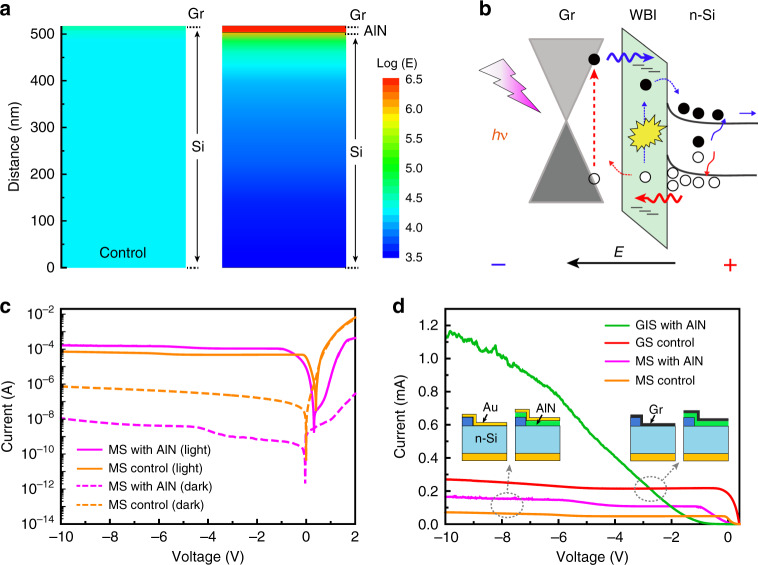
Proposed tunneling mechanism of the GIS devices with comparison to the conventional metal-semiconductor (MS) structure. **a** Simulated electric-field distribution near the junction for the devices of GS and GIS for a comparison (AlN is 15.3 nm and the bias is −10 V). **b** Proposed working mechanisms of the GIS heterojunction photodetector showing the tunneling process from minority carriers of silicon and hot carriers from graphene with impact ionization. **c** The *I-V* curves of MS tunneling photodetector with 15.3-nm AlN comparing with the conventional MS Schottky photodetector under dark and light illumination (365 nm and 12.2 mW cm^−2^). **d** Comparison of the photocurrent for the MS and GIS/GS devices with and without AlN tunneling layer under the reverse bias. The incident light was 365 nm and in a power of 12.2 mW cm^−2^.

Here, the significantly enhanced photo-detection performance is proposed to be originated from two aspects. Firstly, due to the high resistivity and thin thickness of the insulating layer, the imposed reverse voltage is mainly applied to the insulator, causing the high electric field in the region and enhanced energy band bending for silicon and AlN. As a result, the impact ionization would happen during the carries’ tunneling with high kinetics^32^. The simulated electric-field distribution near the junction (Fig. 4a and Fig. S11) clearly shows the significantly enhanced electric field intensity about 6.2 × 10^6^ V cm^−1^ across the insulating layer (under −10 V bias), showing about two order enhancement comparing with the conventional structure (~4.1 × 10^4^ V cm^−1^) without insulating layer (Fig. S11). As for the incident power-dependent responsivity shown in Fig. 3c, the change of electric field within the insulating layer due to the photo-generated carriers’ accumulation was considered to be the main reason^31^, as discussed in Fig. S12.

Secondly, the enhanced photo-detection should have benefitted from the tunneling effect of the hot carriers in graphene, as illustrated in Fig. 4b. Under the high electric field driving the hot carriers (electrons) from graphene can easily overcome the barriers to tunnel through the AlN insulating layer with the possible impact ionization beside the generally considered minority (holes) from the silicon side. In order to demonstrate this, we have fabricated the conventional devices using the thin metal layer (10-nm Au film) as the transparent conductive electrodes for comparison, as shown in Fig. 4c and d. Similar as the GIS devices with AlN layer, the MS device also shows obviously decreased dark current and enhanced photocurrent. Obviously, multiplication has happened on the MS device with AlN tunneling layer as expected. However, much low photocurrent enhancement about 2.3-times was obtained on the MS devices with AlN compared to the ~4.5-times for the GIS devices, while with comparison to each control device without AlN tunneling layer (Fig. 4d). Considering the tunneling carriers should mainly come from the minority of silicon besides the limited thermionic-field emission under reverse bias for the MS device with insolating layer, the additional enhancement for GIS device should originate from the possibly existed multiplication of hot carriers from graphene during the tunneling.

In addition, the specific defects in AlN formed during the ALD deposition^38,44^, were believed to be responsible for the multiplication of the transported carriers due to the significantly increased tunneling current via the mechanism of trap-assisted tunneling^33,45^. Generally, the substitutional oxygen for nitrogen (O_N_) and aluminum vacancy (V_Al_) are the dominated defects in the ALD prepared AlN films^38^, and would form the shallow level defects with the corresponding energy level about 0.8 and 1.0 eV, respectively. As a result, these point defects could further fascinate the hot carriers’ tunneling from graphene as well as the minority from silicon to generate additional photocurrent with multiplication.

**Fig. 5:**
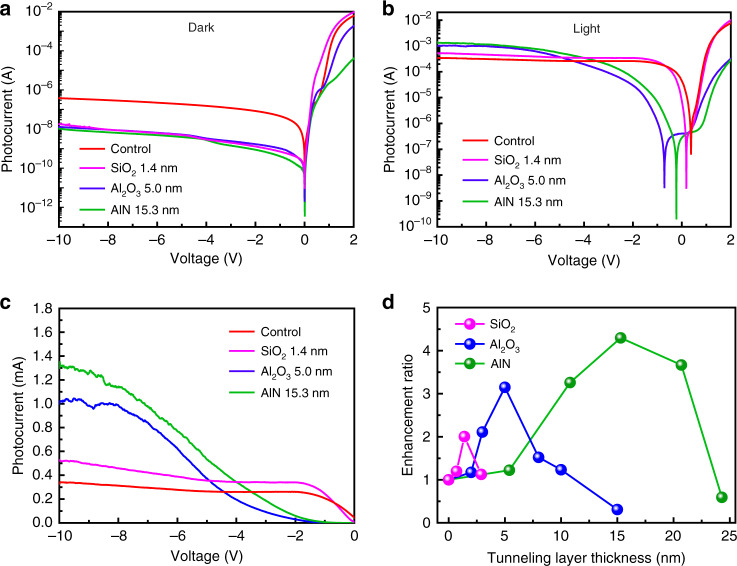
Comparison of the photo-response for the GIS devices using different insulating materials. *I-V* characterizations of the three types of graphene/insulator (SiO_2_, Al_2_O_3_, and AlN)/silicon (GIS) photodetectors under **a** dark and **b** 365-nm light illumination (12.2 mW cm^−2^), with the comparison to the conventional GS heterojunction photodetector. The thickness of the insulators used in (**a**–**c**) for the three types of GIS photodetectors all have been optimized to the best-performed one. **c** The enlarged illustration of (**b**) in the reverse bias region. **d** Tunneling layer thickness-dependent photocurrent enhancement for GIS devices with the three kinds of insulator materials.

Obviously, the band structure of the interfacial insulating layer should be the dominate factors for the tunneling as well as the current multiplication processes. In order to demonstrate the universality of this proposed tunneling mechanism, other commonly used interfacial insulating materials, the native oxidized SiO_2_^29,34^ and Al_2_O_3_^32,46^, also have been employed as the tunneling layer in the GIS heterostructure as comparisons (Figs. S13 and S14). It can be seen that all of the three kinds of insulator in their individually optimized thicknesses (15.3 nm, 5.0 nm, and 1.4 nm for AlN, Al_2_O_3_ and SiO_2_, respectively) can effectively suppress the dark current from 10^−6^ A to the almost same level about 10^−8^ A under the −10 V bias, as shown in Fig. 5a. Figure 5b shows the *I-V* curves of the corresponding three types of GIS devices with Al_2_O_3_, SiO_2_ and AlN tunneling layer under the 365 nm illumination (12.2 mW cm^−2^), comparing with the control device without tunneling layer. From the enlarged view of the photocurrent in the reverse bias region shown in Fig. 5c, it can be seen that all of the tunneling structures show an obvious photo-induced multiplication effect and have significantly enhanced photocurrent compared to those in the control device under the bias of −10 V. Among them, the AlN-tunneling layer achieved the best-performed photo-response and current enhancement followed by the Al_2_O_3_ and SiO_2_ (Fig. 5d). For Al_2_O_3_ film, due to the larger bandgap formed barriers for tunneling and the usual satisfied crystallinity with less defects for the films fabricated by ALD, less thickness was needed for this kind of insulating material. While for the native oxidized SiO_2_ film, the ideal dense and insulation properties of it make the fabricated devices need much critical thickness in nanoscale (<2 nm). Also, the limited thickness strongly decreased the possibility of impact ionization during the tunneling process, thus not suitable for the detection enhancement. Therefore, appropriate band gap and material quality in the tunneling layer, that is well-engineered band structure and SBH are critical for achieving satisfied performances in the GIS tunneling structure.

The dynamic response in the GIS devices also has been investigated at a bias of −10 V and under 365-nm light illumination, as shown in Fig. S15. The measured response time for the rise time (*t*_ON_) is 1.9 ms and the decay time (*t*_OFF_) is 1.4 ms. Different from the carrier drift-diffusion process in the conventional graphene/n-Si photodetector, with the employment of the insulating layer a faster optical response (1.9 ms vs 2.3 ms in GS structure) was realized due to this photo-assisted tunneling process at a higher electric field. Here, the possibly existed interface trapping defects due to the wet-transferring should have limited the response dynamics for the devices^9^. Furthermore, a good environmental stability was also revealed in the fabricated GIS photodetectors with the detection performance remaining nearly unchanged even after being stored in ambient air for 128 days, as shown in Fig. S16.

## Discussion

In this work, a graphene/insulating layer/silicon heterostructure was proposed and fabricated based on energy-band engineering to realize the enhanced impact ionization for photo-detection, besides the simultaneous strong restraint of dark current. Theoretical and experimental results indicated that the tunneling process generated novel impact ionization within the engineered AlN-insulating layer both for hot carries from graphene and minority carriers from silicon contributed to this obvious photogain enhancement. Subsequently, a champion responsivity for the photodetectors reached a relatively stable value of ~1.03 A W^−1^ at a reverse bias of −10 V under the typical UV detection wavelength (365 nm), showing the great potential applications in sensing with 4.20 times enhancement comparing with the conventional GS photodetectors and 7.16-times increment comparing with the typical commercial silicon PIN photodetectors. The peak responsivity was also achieved with 3.96 A W^−1^ at wavelength of 850 nm, presenting about 5.8 times photogain improvement. Considering the low-cost, high performance and silicon integrability, of this kind of GS tunneling heterojunction photodetectors show great potential applications in communication and sensing.

## Materials and methods

### Device fabrication

Double polished n-type silicon wafers with 300-nm SiO_2_ layer were purchased from MTI Co. with a parameter of 0.5 mm-thick and resistivity of 2–4 Ω cm. After standard cleaning, 3 mm × 3 mm silicon windows were exposed in the wafer by photolithography and etched by a buffered oxidizing etching (BOE) solution. Then, after the removal of the photoresist by acetone and the wafer dying, the WBI layer was deposited by ALD or in situ oxidation. Subsequently, graphene was transferred to the patterned window of the device to make the top electrode and the photosensitive region conductive. Considering the sheet conductivity and optical transmittance, the 3–5 layers’ graphene were used in this work which was grown on copper foils (ACS Material) and purchased from Nanjing XFNANO Materials Tech Co., Ltd. Electrodes containing 5-nm Ti and 100-nm Au for both sides were prepared by magnetron sputtering, during which the active area was protected by a mask. Finally, the wafer was scribed into devices with size of 8 mm × 8 mm by laser scribing. The insulating AlN and Al_2_O_3_ films are grown by ALD (Beneq TFS 200). The AlN films was grown by the reaction of Trimethyl Aluminum (TMA) source and NH_3_ source at a temperature of 380 °C while Al_2_O_3_ films is grown by trimethyl aluminium (TMA) source and H_2_O source at a temperature of 200 °C. The details of growth process are shown in Fig. S3 and Table S1. SiO_2_ layer was grown by O_2_ plasma-assisted oxidation (Alpha Q150), and the thickness is controlled by adjusting the O_2_ plasma treatment durations. To fabricate the MS devices, a thin Au layer about 10 nm was used as the transparent conductive electrodes for the replacement of graphene.

### Characterization

The crystal quality of silicon substrate and AlN films was analyzed by XRD (Rigaku IV). The surface morphology was studied by a field emission SEM (Hitachi S-4800). Transmission and absorption spectra were collected by Varian Cary 5000 UV–Vis–NIR spectrophotometer. TEM image and EDX elemental mappings were measured on transmission electron microscope (JEOL JEM-F200). The photocurrent and *I-V* characteristics of the devices were measured using Keithley 2400 Source Meter equipped with a room-temperature probe station and LED light sources. The dark current was analyzed on semiconductor analyser (Keithley SCS-4200), and the response time is measured on oscilloscope (Tektronix TBS-1102). The native doping type and carriers’ concentration for the ALD deposited AlN has been characterized by the Hall effect using a Hall Effect Measurement Systems (HMS-7000, Ecopia) at room temperature.

### Theoretical simulation

The electric field distribution for the GS and GIS device structures under dark condition was simulated using a commercial finite element analysis and modeling software (APSYS, Crosslight Software Inc.) which was based on basic drift and diffusion model. A 2D simplified device model with width of 3 μm was used for the simulation, in which consisted of 15.3-nm thick AlN and 65-μm n-silicon (as schematically shown in Figure S11) for the GIS structure and without AlN for the control GS structure. The doping concentration for the n-silicon was 1 × 10^20^ cm^−3^, and 8.1 × 10^15^ cm^−3^ for the n-type AlN layer which has been experimentally measured by the hall effect. The transparent front Schottky contact with barrier height about 2.2 eV was set as the graphene electrode for simplify and a typical bottom Ohmic contact was used back electrode.

## Supplementary information

Surpporting information
