# Supplementary material for: Engineered tunneling layer with enhanced impact ionization for detection improvement in graphene/silicon heterojunction photodetectors
Source: Light Sci Appl. 2021 May 31;10:113. doi: 10.1038/s41377-021-00553-2 (PMC8167175; doi:10.1038/s41377-021-00553-2)
Supplement: Supplementary file 1 — Surpporting information [file 41377_2021_553_MOESM1_ESM.docx]

Supplementary Information for

Engineered tunneling layer with enhanced impact ionization for detection improvement in graphene/ silicon heterojunction photodetectors

Jun Yin,^*a^ Lian Liu,^a^ Yashu Zang,^b^ Anni Ying,^a^ Wenjie Hui,^a^ Shusen Jiang,^a^ Chunquan Zhang,^a^ Tzuyi Yang,^c^ Yu-Lun Chueh,^c^ Jing Li,^*a^ and Junyong Kang^a^

^a.^ Collaborative Innovation Center for Optoelectronic Semiconductors and Efficient Devices, Pen-Tung Sah Institute of Micro-Nano Science and Technology/ Department of Physics, Xiamen University, Xiamen 361005, China.

^b.^ San'an Optoelectronics Co., Ltd., Xiamen 361005, China.

^c.^ Department of Materials Science and Engineering, Tsing Hua University, Hsinchu 30013, China.

E-mail: [jyin@xmu.edu.cn](mailto:jyin@xmu.edu.cn); [lijing@xmu.edu.cn](mailto:lijing@xmu.edu.cn)


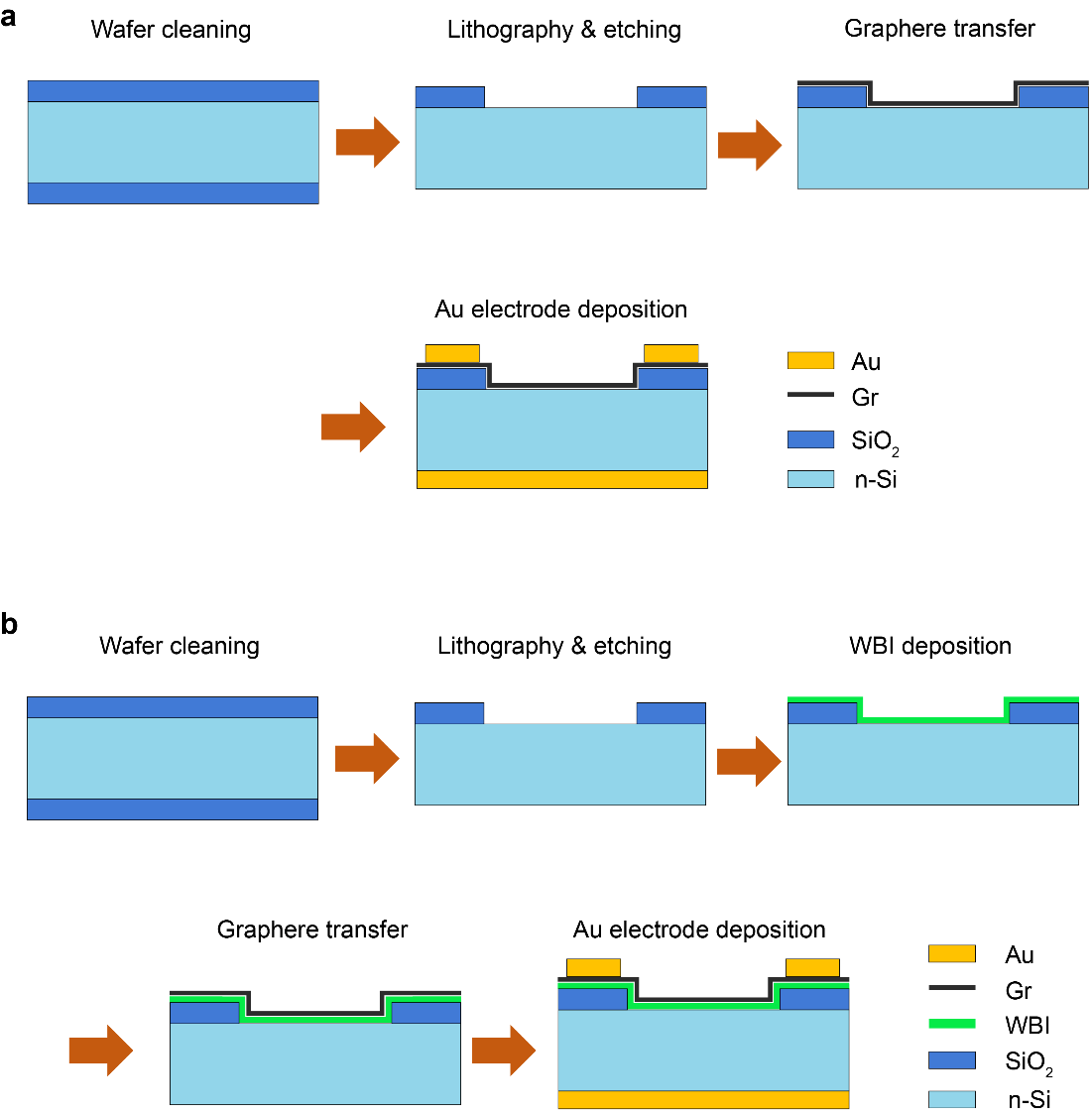


**Figure S1.** Schematic diagram of the preparation process of the (a) conventional graphene/n-Si heterojunction photodetector and (b) graphene/AlN/n-Si photodetector.


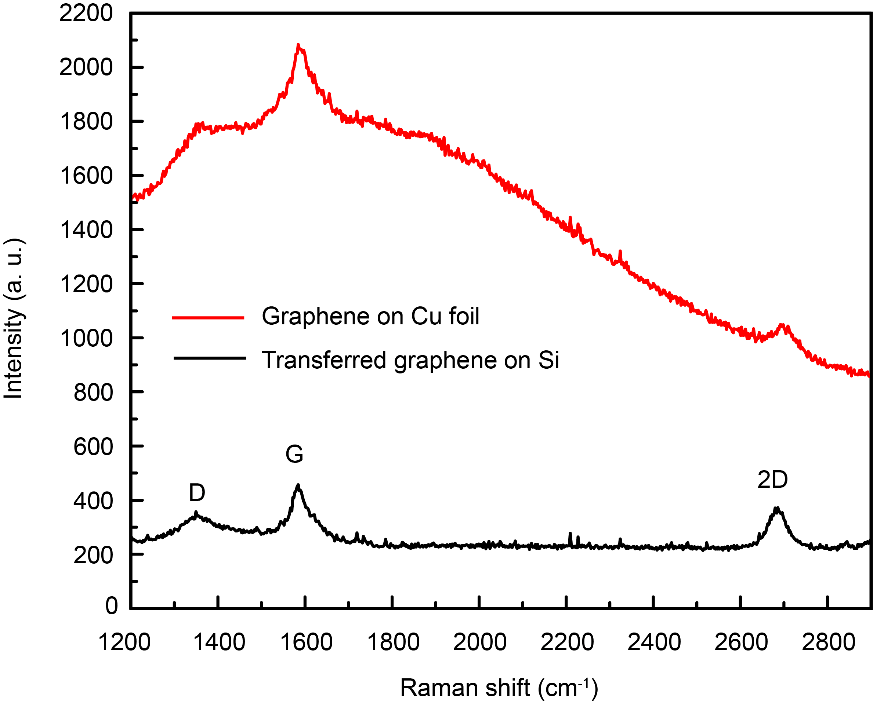


**Figure S2.** Raman spectra of the pristine 3~5 layers’ graphene on Cu foil and transferred graphene on silicon substrate. It can be seen that the signature D, G and 2D peaks located at 1350, 1582 and 2700 cm^-1^ have been resolved for the transferred graphene, which show the typical characteristics of the few layers graphene with the respect to the intensity of 2D band relative to G band (*I*_2D_/*I*_G_≈0.67). Also, the no obvious change of the signature peaks for the transferred graphene as that in the pristine graphene grown on Cu foil besides of the typical strong fluorescence background indicates the well maintained high quality of the graphene.


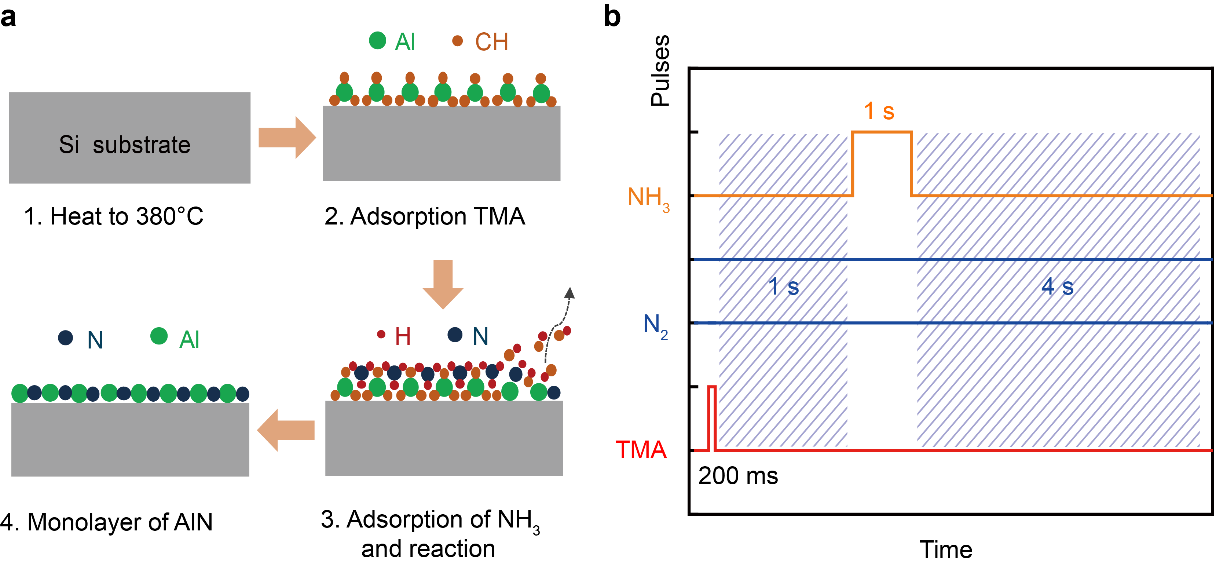


**Figure S3.** (a) Schematic illustration of the growth mechanism of the AlN films by atomic layer deposition (ALD) using Ammonia (NH_3_) and Trimethyl aluminum(TMA) as reaction sources, and Si as substrates. (b) Growth parameters in one ALD cycle in this work.

| Materials | ALD Cycles | Thickness (nm) | Details |
| --- | --- | --- | --- |
| AlN | 60 | 5.4 | Temperature: 380℃  Source: NH_3_, TMA  Thickness: 0.09 nm/cycle |
|  | 120 | 10.8 |  |
|  | 170 | 15.3 |  |
|  | 230 | 20.7 |  |
|  | 270 | 24.3 |  |
| Al_2_O_3_ | 30 | 3 | Temperature: 200℃  Source: H_2_O, TMA  Thickness: 0.1 nm/cycle |
|  | 50 | 5 |  |
|  | 80 | 8 |  |
|  | 100 | 10 |  |
|  | 150 | 15 |  |

**Table S1.** Thickness and details of AlN and Al_2_O_3_ films in the ALD growth


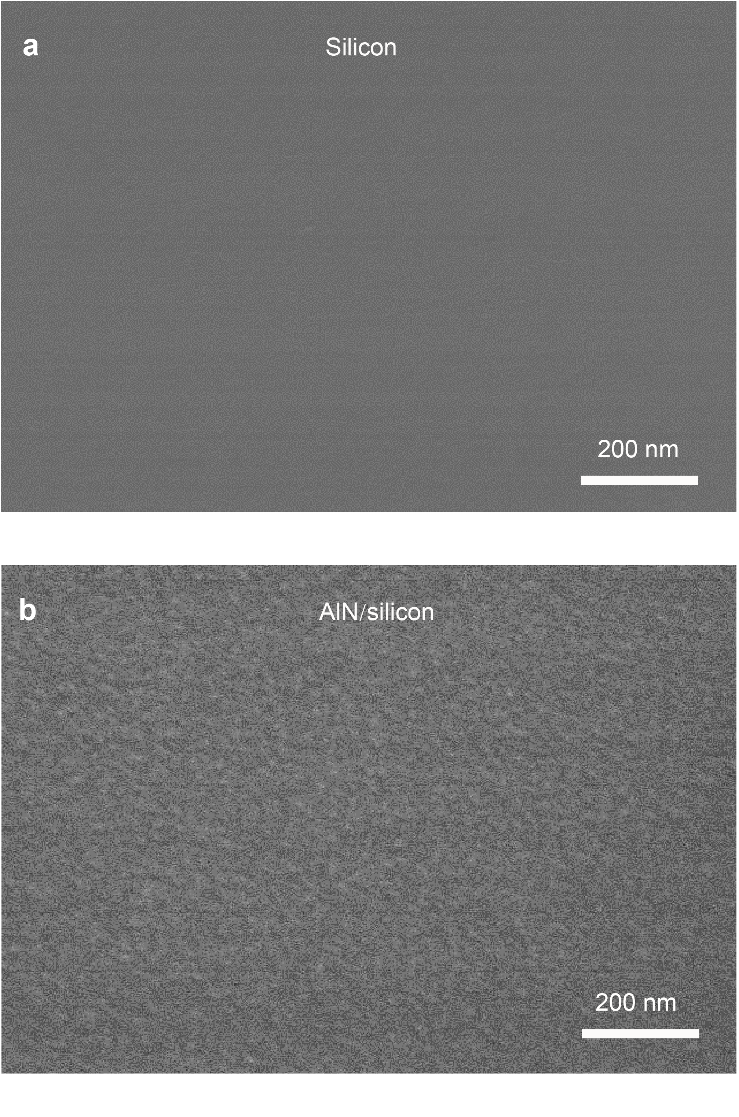


**Figure S4.** SEM images of (a) Si substrate and (b) AlN films with thicknesses 15.3 nm on the silicon substrate.


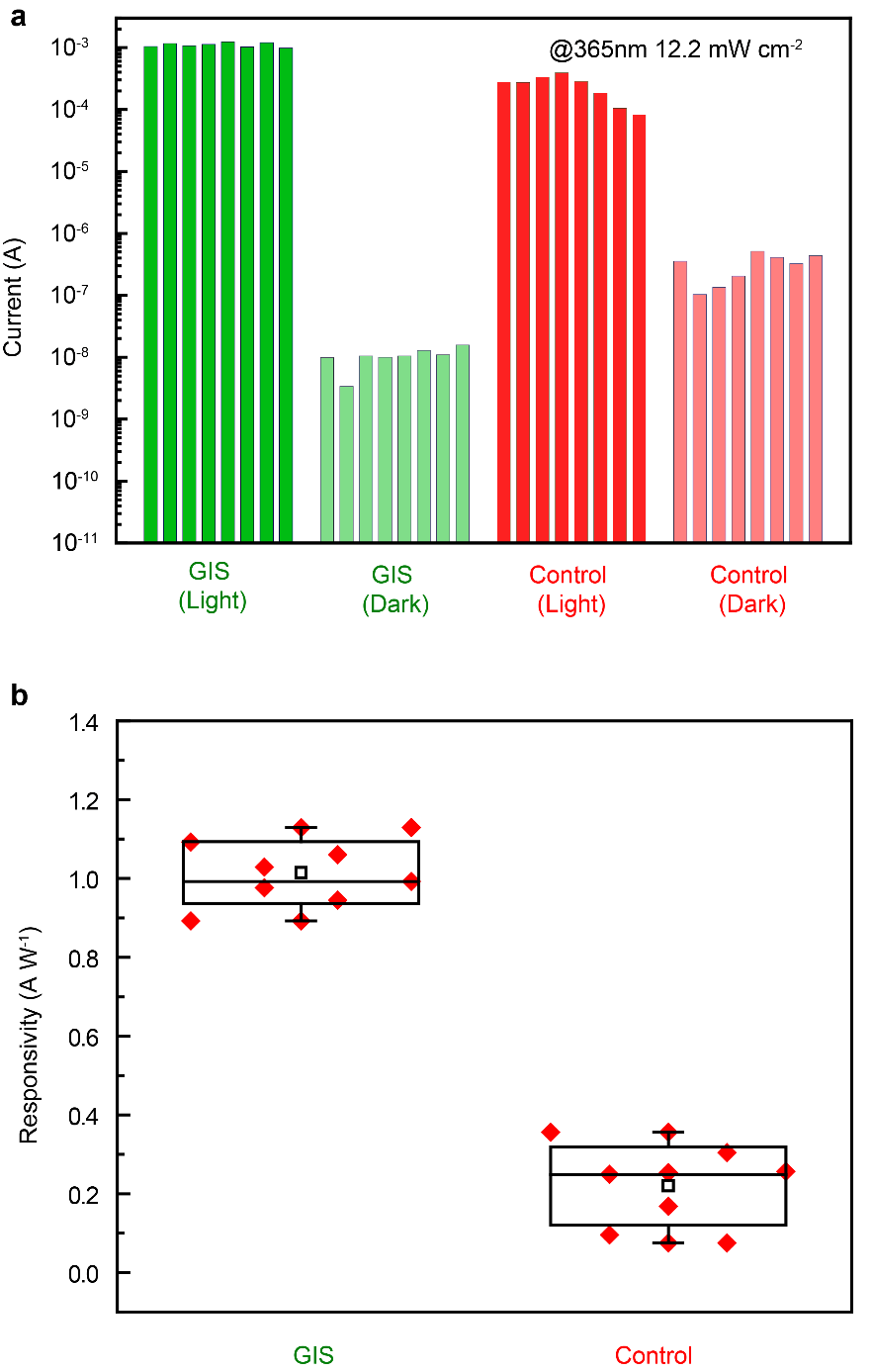


**Figure S5.** (a) Statistics of the photocurrent (365-nm irradiation,12.2 mW cm^-2^) and dark current of the GIS devices (a batch of 8 devices) with comparison to the GS control ones; (b) Responsivity metrics for the GIS and GS devices.


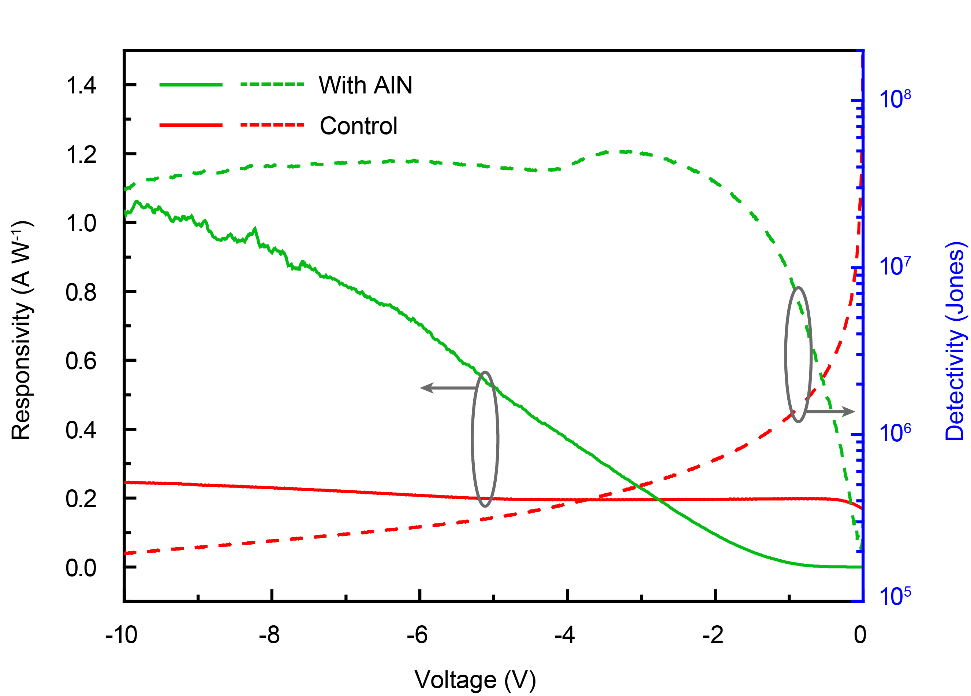


**Figure S6.** Reverse bias dependent responsivity and specific detectivity for the GIS photodetectors with 15.3-nm AlN layer under the 365 nm irradiation. It can be seen that as the reverse bias (electric-field) increased, the responsivity also increased accordingly which is similar as that in the previous report for the AlN avalanche photodetectors with impact ionization process.^1^


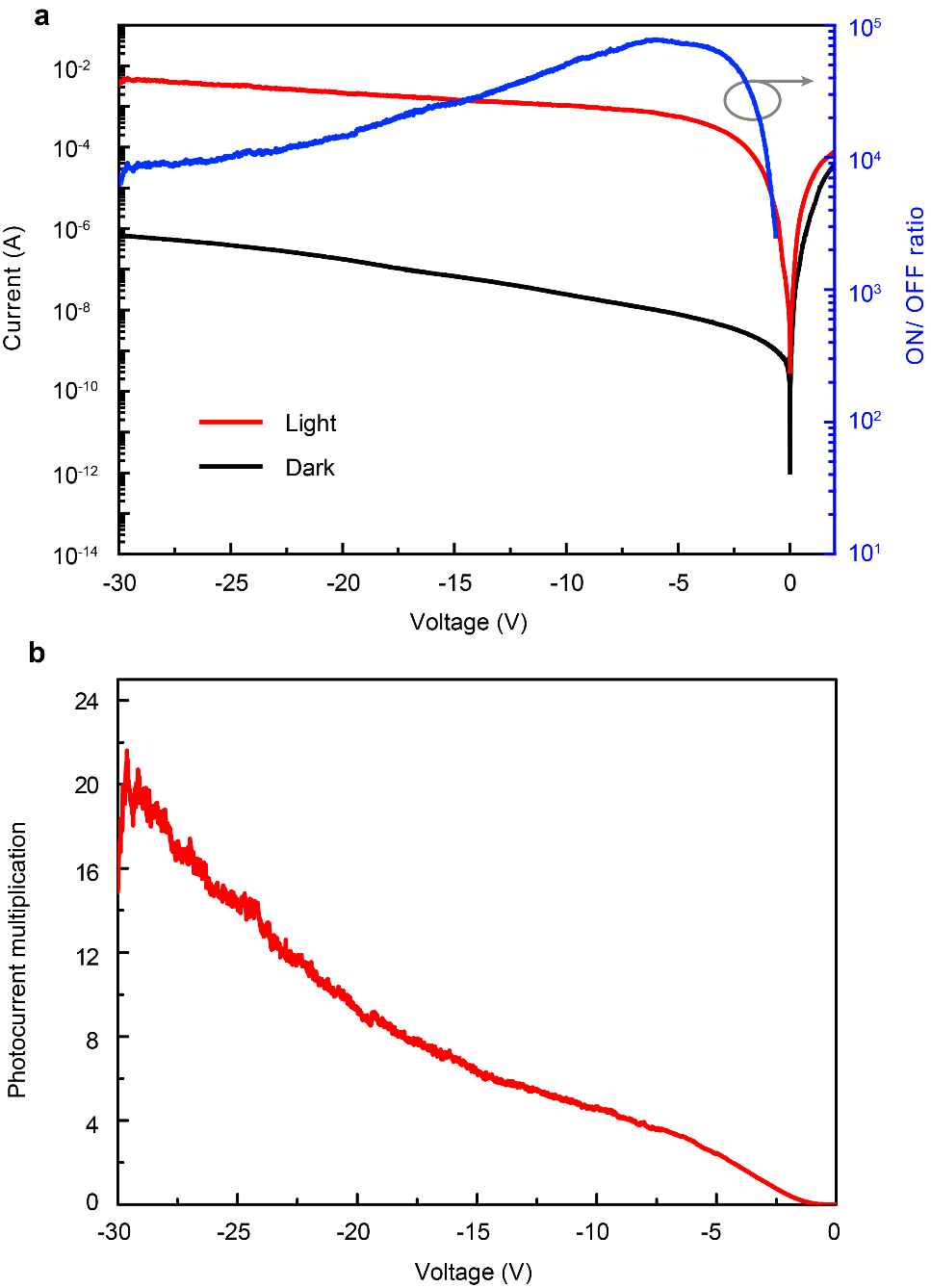


**Figure S7.** (a) *I-V* characterizations and ON/OFF ratio of the GIS photodetector with 15.3-nm AlN comparing with the control device under dark and light illumination (365 nm and 12.2 mW cm^-2^) with a bias from -30 V to 2 V. The results also indicated that the GIS devices with tunneling layer still can work well even under high bias of -30 V. (b) Calculated multiplication (gain) of the GIS device as a function of the reverse bias voltage under 365-nm illumination.


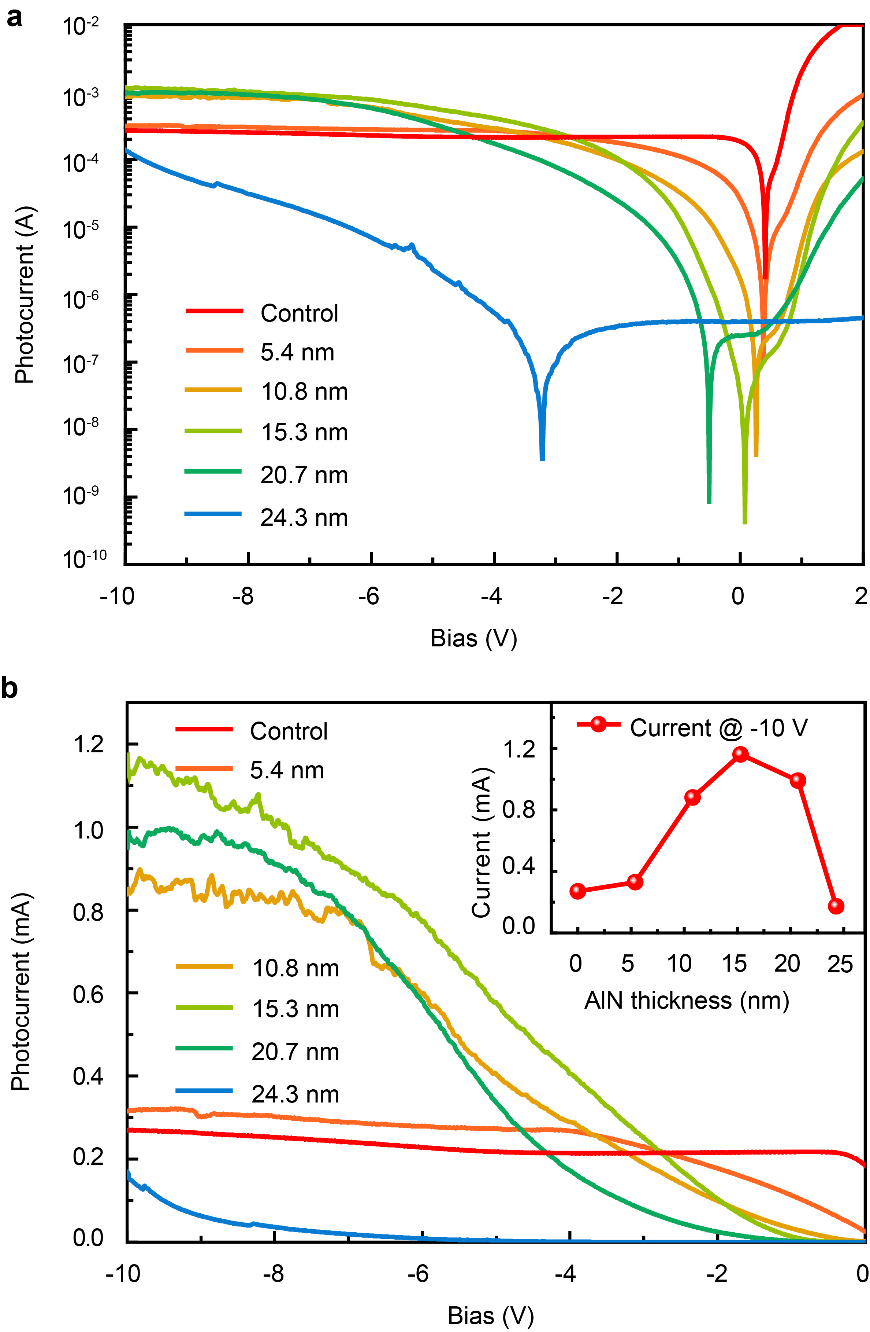


**Figure S8.** (a) *I-V* curves of the GIS photodetectors with different thicknesses of AlN layer under the 365 nm illumination (12.2 mW cm^-2^). (b) The enlarged view of the photocurrent response under reverse bias with the statistics of the current values under -10 V shown in inset.


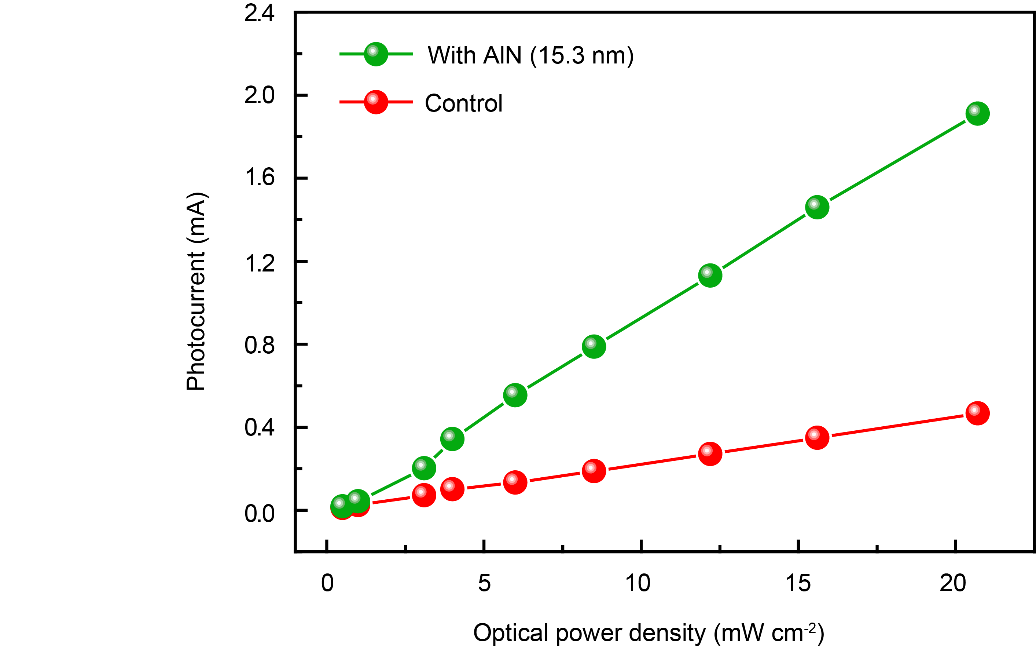


**Figure S9.** Optical power dependent photocurrent in the GIS tunneling photodetector with the comparison to the control device without tunneling layer under 365 nm illumination at a bias of -10 V.


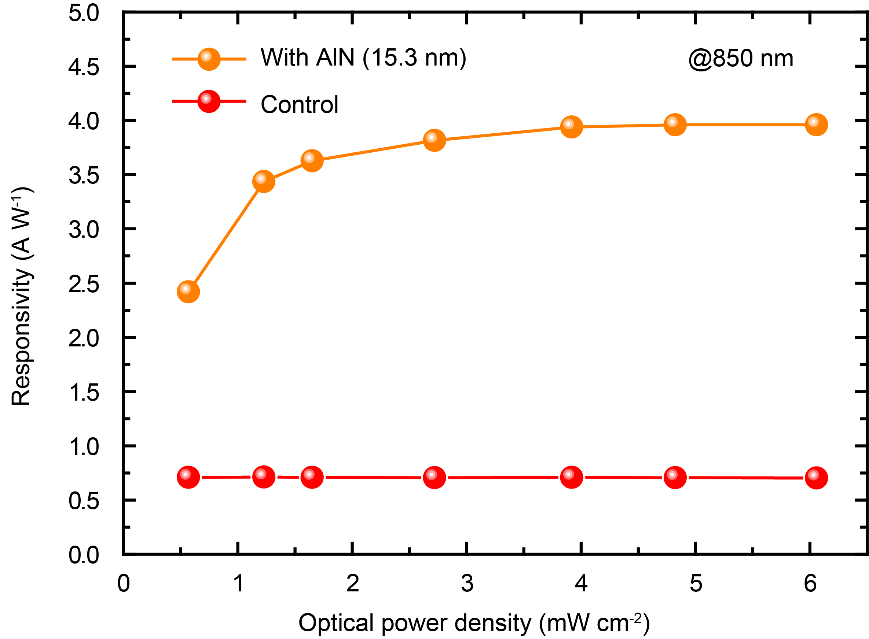


**Figure S10.** Responsivity for the GIS tunneling photodetector with comparison to the control device without tunneling layer under 850 nm illumination at a bias of -10 V.

**Table S2.** Recent progress in graphene/silicon heterostructure photodetector with an interface insulating layer.

| Device structure | Detecting mode | Area (mm^2^) | Working bias (V) | R (A W^-1^) | Detectivity (Jones) | Ref. |
| --- | --- | --- | --- | --- | --- | --- |
| Gr/n-Si | Schottky | 0.005 | -2 | 0.435@850 nm | 7.69×10^9^ | 2 |
| Gr/SiO_2_/n-Si | Schottky | 10 | 0 | 0.73@890 nm | 4.08×10^13^ | 3 |
| Gr/SiO_2_/p-Si | Schottky | 1.44 | -5 | 5.5@458 nm | 2.35×10^10^ | 4 |
| Gr/p-Si | Schottky | 0.25 | 0 | 0.2@365 nm | 1.6×10^13^ | 5 |
| Gr/Al_2_O_3_/n-Si | Tunneling | -- | -20 | 0.75@658 nm | 3.1 × 10^12^ | 6 |
| Gr/AlN/n-Si | Tunneling | 9.0 | -10 | 1.03@365 nm 3.96@850 nm | 2.94×10^7^(365 nm) 1.13×10^8^(850 nm) | This work |

**
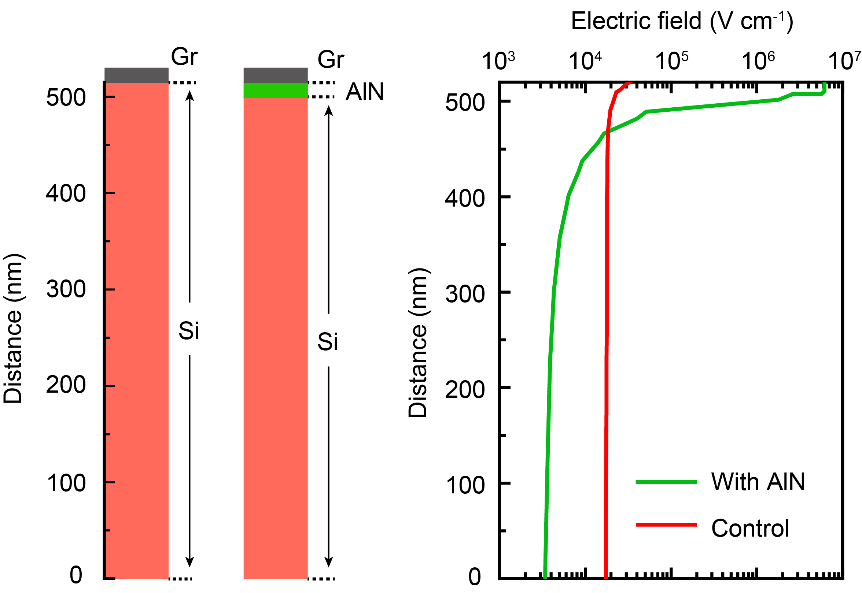
**

**Figure S11.** Schematical illustration of device structure near the heterojunction in the simulation. The simulated electric filed intensity near the heterojunction was shown in the figure for the GPD devices with and without AlN tunneling layer for a comparison.


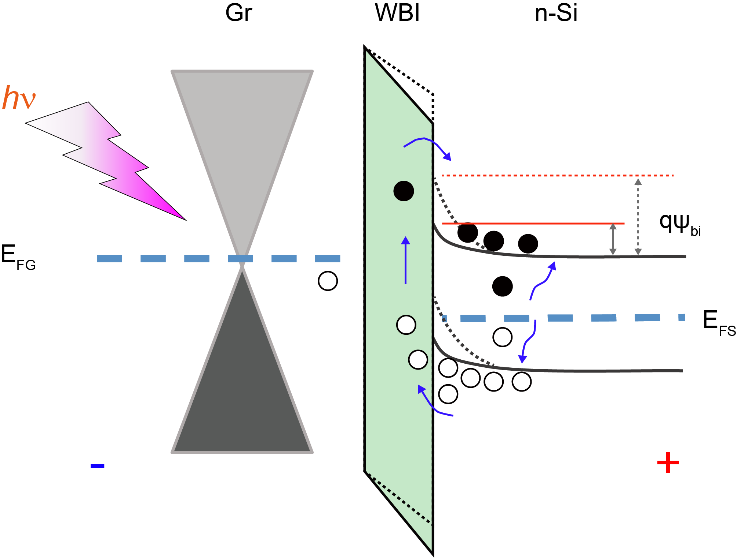


**Figure S12.** Energy-band diagram of the GIS tunneling structure under low (dash line) and high (solid line) light intensity irradiation, showing the typical thermodynamic equilibrium regime and non-equilibrium stationary depletion regimes. Under the weak light illumination, the band alignment is mainly in the equilibrium state (dash line), the charges’ accumulation in insulator-semiconductor interface can be ignored. While if intensifying the incident light (solid line), sufficient charges’ accumulation would increase the electric field in the insulating layer, resulting in higher ionization rate during the tunneling process and the subsequent further enhanced photo responsivity. However, as the optical power density increasing to a certain value, due to the shielding effect of the accumulated charges, the total equilibrium band bending *qψ_bi_* would be relatively small and the negative bias on the space-charge region would decrease. With the decrease of the negative bias on the space-charge region, the carriers’ recombination rate would rise, leading to a relative stable value of photo responsivity as the optical power density continues to increase. Thus, a photodetection linearity region was achieved when the incident optical power increased to mW level.


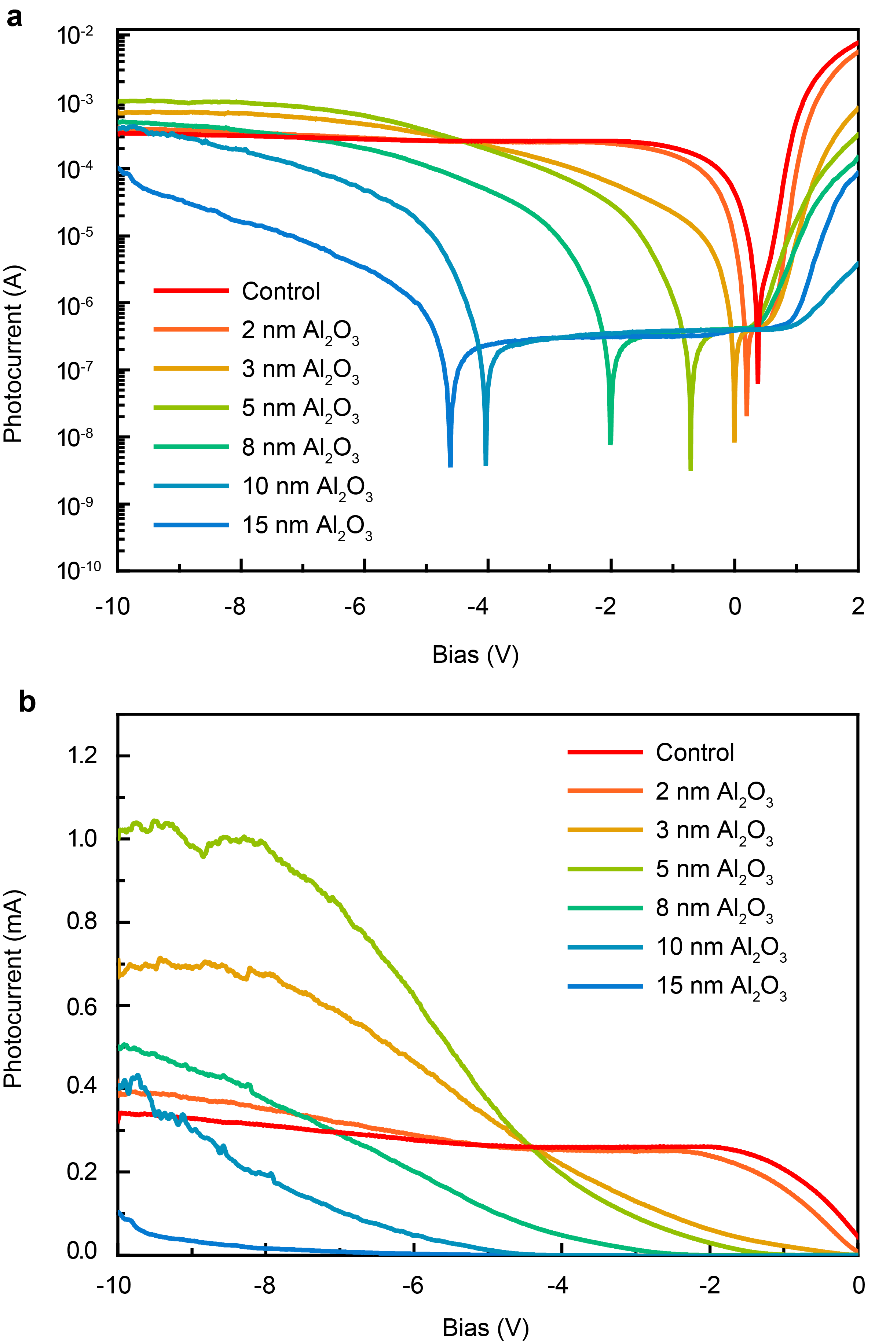


**Figure S13.** (a) Photocurrent-voltage characterizations of the graphene/Al_2_O_3_/n-Si photodetectors under 365 nm illumination (12.2 mW cm^-2^). (b) The enlarged view of (a).


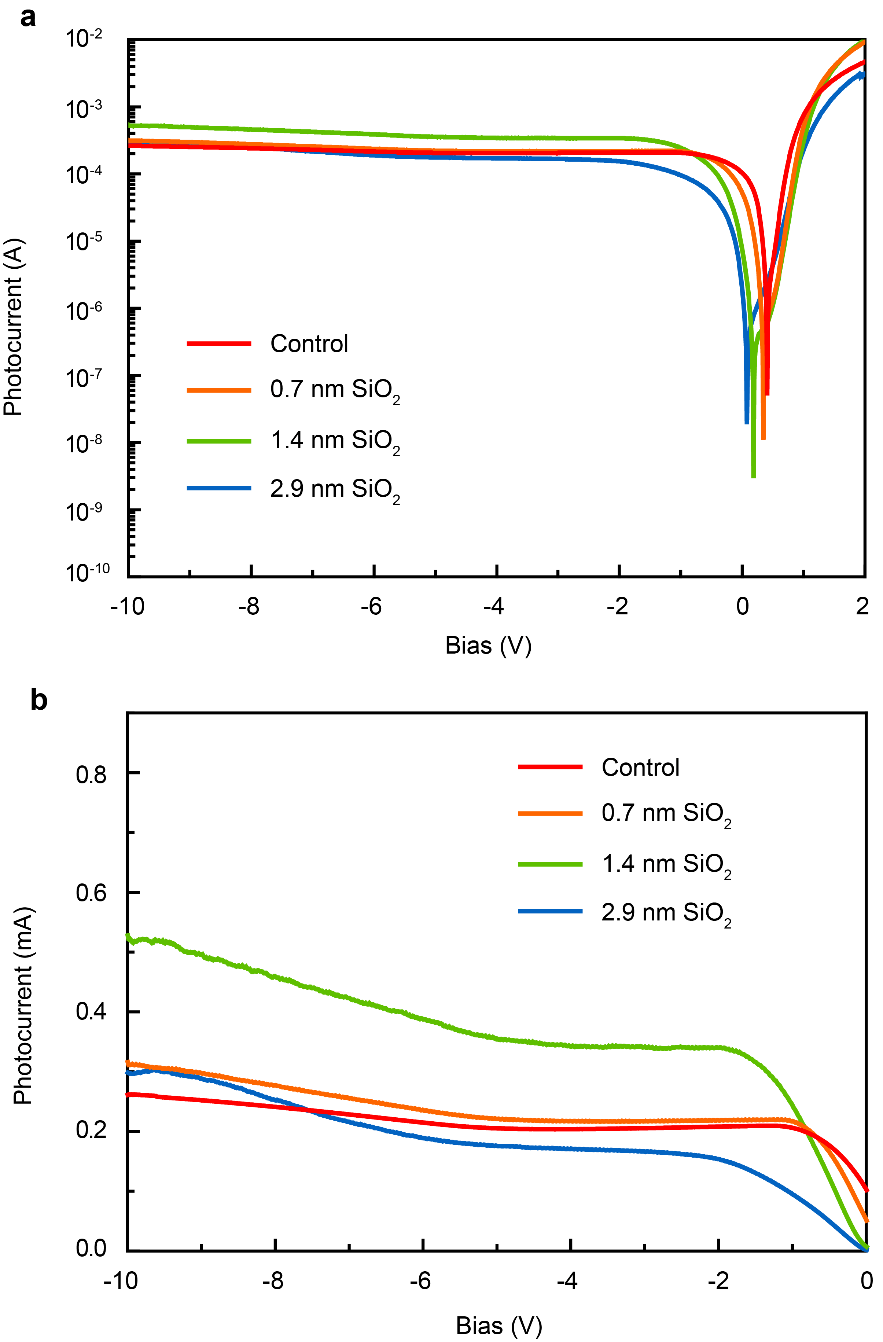


**Figure S14.** (a) Photocurrent-voltage characterizations of the graphene/SiO_2_/n-Si photodetectors under 365 nm illumination (12.2 mW cm^-2^). (b) The enlarged view of (a).


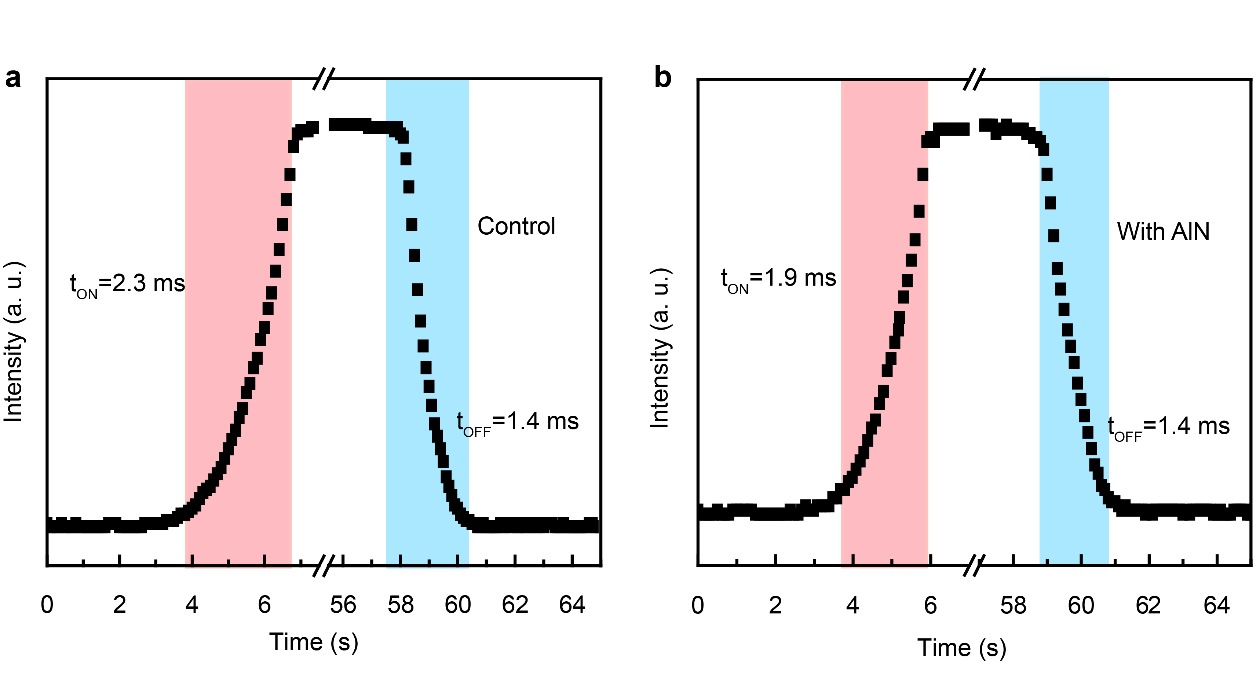


**Figure S15.** Response time of the GIS tunneling photodetector with15.3 nm AlN films under 365 nm illumination at a bias of -10 V.

**
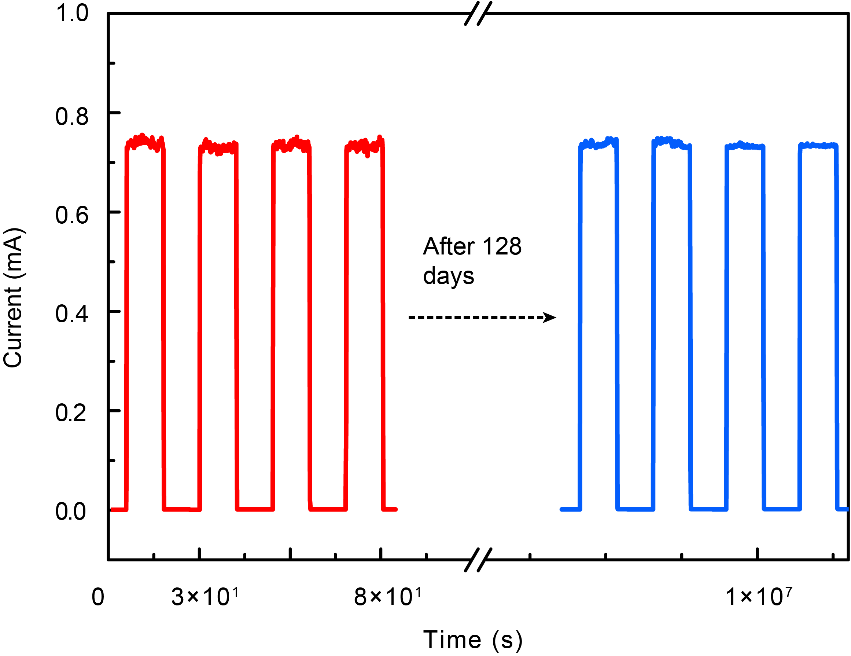
**

**Figure S16.** Long-term stability measurement of the GIS tunneling photodetector which was stored in an atmospheric environment and room-temperature.

**REFERENCE**

1. Dahal, R.; Tahtamouni, T. M.; Lin, J. Y.; Jiang, H. X., AlN avalanche photodetectors. *Appl. Phys. Lett.* **2007**, 91(24), 243503.

2. An, X.; Liu, F.; Jung, Y. J.; Kar, S., Tunable graphene-silicon heterojunctions for ultrasensitive photodetection. *Nano Lett.* **2013**, 13 (3), 909-16.

3. Li, X.; Zhu, M.; Du, M.; Lv, Z.; Zhang, L.; Li, Y.; Yang, Y.; Yang, T.; Li, X.; Wang, K.; Zhu, H.; Fang, Y., High Detectivity Graphene-Silicon Heterojunction Photodetector. *Small.* **2016**, 12 (5), 595-601.

4. Park, H.-K.; Choi, J., High Responsivity and Detectivity Graphene-Silicon Majority Carrier Tunneling Photodiodes with a Thin Native Oxide Layer. *ACS Photonics*. **2018**, 5 (7), 2895-2903.

5. Wan, X.; Xu, Y.; Guo, H.; Shehzad, K.; Ali, A.; Liu, Y.; Yang, J.; Dai, D.; Lin, C.; Liu, L.; Cheng, H.; Wang, F.; Wang, X.; Lu, H.; Hu, W.; Pi, X.; Dan, Y.; Luo, J.; Hasan, T.; Duan, X.; Li, X.; Xu, J.; Yang, D.; Ren, T.; Yu, B. A self-powered high-performance graphene/silicon ultraviolet photodetector with ultra-shallow junction: breaking the limit of silicon ?. *npj 2D Materials and Applications*, **2017**, 1(1), 1-8.

6. Xu, J.; Liu, T.; Hu, H.; Zhai, Y.; Chen, K.; Chen, N.; Li, C.; Zhang, X.; Design and optimization of tunneling photodetectors based on graphene/Al_2_O_3_/silicon heterostructures. *Nanophotonics*. **2020**, 9(12): 3841-3848.
